# Transmembrane substrates of type three secretion system injectisomes

**DOI:** 10.1099/mic.0.001292

**Published:** 2023-01-27

**Authors:** Camilla Godlee, David W. Holden

**Affiliations:** ^1^​ MRC Centre for Molecular Bacteriology and Infection, Imperial College London, Armstrong Road, London SW7 2AZ, UK; ^†^​ Present address: Department of Infectious Diseases, School of Immunology and Microbial Sciences, King's College London, London, UK

**Keywords:** effector, injectisome, membrane integration, translocon, type three secretion system

## Abstract

The type three secretion system injectisome of Gram-negative bacterial pathogens injects virulence proteins, called effectors, into host cells. Effectors of mammalian pathogens carry out a range of functions enabling bacterial invasion, replication, immune suppression and transmission. The injectisome secretes two translocon proteins that insert into host cell membranes to form a translocon pore, through which effectors are delivered. A subset of effectors also integrate into infected cell membranes, enabling a unique range of biochemical functions. Both translocon proteins and transmembrane effectors avoid cytoplasmic aggregation and integration into the bacterial inner membrane. Translocated transmembrane effectors locate and integrate into the appropriate host membrane. In this review, we focus on transmembrane translocon proteins and effectors of bacterial pathogens of mammals. We discuss what is known about the mechanisms underlying their membrane integration, as well as the functions conferred by the position of injectisome effectors within membranes.

## Introduction

Infection of mammalian hosts by bacterial pathogens often relies on the activity of virulence proteins (effectors) that are delivered into host cells and target a variety of host cellular processes, enabling bacterial invasion, nutrient acquisition and interference with host immune functions. The type three secretion system (T3SS) injectisome, (hereafter referred to as the injectisome) is commonly used by pathogenic Gram-negative bacteria for delivery of effectors across a host cell membrane [1]. For example, the injectisomes of pathogenic *Escherichia coli *, *Yersinia *, *Pseudomonas *, *Vibrio *, *Shigella* and *Salmonella* pathogenicity island (SPI)-1 deliver their effectors across the plasma membrane of infected cells [1]. After invasion of the host cell, *Salmonella* resides within a membrane-bound compartment known as the *Salmonella *-containing vacuole (SCV), and its SPI-2-encoded injectisome delivers effectors across the SCV membrane [2]. *Chlamydia* is an obligate intracellular pathogen that resides within a large membrane-bound vacuole, known as the inclusion body, and its injectisome delivers effectors across this membrane [3].

Injectisome-dependent effector delivery into host cells first requires assembly of the secretion apparatus, which consists of a basal body that spans the inner and outer membrane of the bacterium and a hollow needle that extrudes from the surface of the bacterium [1]. Two transmembrane proteins known as the major and minor translocon, based on their sizes, are then secreted through the needle (Table 1) [1]. In some cases, a hydrophilic protein that forms the needle ‘tip complex’ aids the integration of these translocon proteins into the host cell membrane to form the translocon pore, through which effectors are delivered [4–7].

**Table 1.** Translocon components from different type three secretion system injectisomes

Injectisome	Minor	Major	Chaperone	Hydrophilic tip/filament
Standard	SctB	SctE	-	SctA
* Yersinia enterocolitica *	YopD	YopB	SycD	LcrV
* Pseudomonas aeruginosa *	PopD	PopB	PcrH	PcrV
* Aeromonas hydrophila *	AopD	AopB	AcrH	AcrV
* Shigella *	IpaC	IpaB	IpgC	IpaD
* Salmonella* SPI-1	SipC	SipB	SicA	SipD
* Salmonella* SPI-2	SseD	SseC	SseA	SseB
Pathogenic *Escherichia coli *	EspB	EspD	CesAB	EspA
* Chlamydia *	CopD	CopB	-	CT584
* Vibrio parahaemolyticus* T3SS-2	VopD2	VopB2	-	VopW

The subcellular localization of effectors within the host cell is often important for their functionality [8, 9]. Some localize to the cytoplasm, others enter organelles and some associate with host cell membranes [8]. Since membrane association limits the diffusion of the protein to two dimensions, this is an efficient way to concentrate a protein at a specific cellular location. Additionally, many important cellular processes occur at membranes. Some injectisome effectors associate with membranes after undergoing post-translational lipidation (for example the *Pseudomonas* effector AvrB [10]), or by direct interaction with a host membrane protein or lipid (for example the *Salmonella* effector SteA [11]), or by integrating into the membrane. We refer to this latter group as transmembrane effectors (TMEs) (Table 2) [12].

**Table 2.** Transmembrane effectors and their functions. This table only contains transmembrane effectors with well-defined functions that are discussed in the review

Injectisome	Effector	Function
* Salmonella* SPI-2	SteD	Reduction in mMHCII, CD97 and CD86 surface levels
* Salmonella* SPI-2	SseF	Close association of *Salmonella *-containing vacuole with Golgi
* Salmonella* SPI-2	SseG	Close association of *Salmonella *-containing vacuole with Golgi
* Escherichia coli *	Tir	Receptor for *Escherichia coli* attachment
* Escherichia coli *	EspZ	Regulates effector translocation
* Escherichia coli *	NleA	Reduces protein secretion
* Chlamydia *	IncA	Homotypic fusion of inclusion membrane
* Chlamydia *	IncD	Membrane contact site with the endoplasmic reticulum
* Chlamydia *	IncV	Membrane contact site with the endoplasmic reticulum
* Chlamydia *	Cpn0585	Recruitment of Rabs to the inclusion membrane
* Chlamydia *	CT229	Recruitment of Rabs to the inclusion membrane
* Chlamydia *	IncE	Recruitment of SNX5/6 to the inclusion membrane
* Vibrio parahaemolyticus* T3SS-1	VopQ	Membrane disruption and ion release

The mechanisms by which translocon components and TMEs, collectively referred to here as injectisome TM substrates, integrate into membranes are not well understood. In this review we focus on TM substrates of bacterial pathogens of mammals. We describe the specific features that allow them to be delivered into host cells and what is known of the mechanisms that underlie their targeting and integration into host cell membranes. We also discuss the biochemical functions that are conferred by injectisome TM substrates within the membrane.

## Avoiding aggregation and integration into the bacterial inner membrane

Hydrophobic TM proteins tend to aggregate in solution. Bacterial inner membrane proteins avoid aggregation in the bacterial cytoplasm through co-translational membrane insertion. Their hydrophobic TM regions are recognized upon exit from ribosomes by the signal recognition particle (SRP) complex [13], which delivers them to the SecYEG translocon [14], thus avoiding contact with the cytoplasm. Therefore, injectisome TM substrates must avoid not only aggregation in the bacterial cytoplasm but also integration into the bacterial inner membrane through recognition by the SRP pathway during translation.

Many injectisome proteins interact with chaperones in the bacterial cytoplasm. Class I chaperones interact with effectors, while class II chaperones interact with translocon proteins. These chaperones dissociate from their cognate substrates upon entry into the injectisome and are not themselves delivered into host cells [15, 16]. In the bacterial cytoplasm, class I chaperones prevent premature folding of effectors, which helps to retain them in a secretion-competent state [15].

Class II chaperones contain tetratricopeptide repeats and form a helical curved structure [17]. The major and minor translocon components from the injectisomes of *Yersinia enterocolitica *, *Pseudomonas aeruginosa *, *Aeromonas hydrophila *, *Shigella* and *Salmonella* SPI-1 contain a consensus P/VXLXXP chaperone-binding motif known as the ‘molecular anchor’, N-terminal to the TM region, with which they bind to the concave face of the curved structure of a common class II chaperone (Table 1) [7, 17–19]. However, the *Salmonella* SPI-2, *E. coli* and *Chlamydia* translocon components do not contain this motif. The translocon fragments used for characterization of the molecular anchor did not include the TM regions, so it is impossible to determine from these studies whether class II chaperones also interact and protect the TM regions of translocon components. However, the solved structure of a larger segment of AopB, the major translocon of *A. hydrophila *, in complex with its chaperone AcrH, shows additional extensive interactions between AcrH and the TM regions of AopB [20]. The more hydrophobic TM region of AopB is positioned between AcrH and the less hydrophobic TM region of AopB. This arrangement could protect the more hydrophobic regions of the translocon from aggregation in the bacterial cytoplasm. Given the homologous nature of the translocon proteins and their chaperones between species, it is plausible that protection of translocon TM regions by their cognate chaperones applies to other injectisomes. In agreement with this, otherwise insoluble translocon proteins become soluble when co-purified with their chaperones [21].

Despite a lack of primary sequence similarity, class I chaperones share structural similarity and form horseshoe-like homodimers with an α–β fold [22]. Effectors, both with and without TM regions, interact with their cognate chaperones through an N-terminal chaperone-binding domain (CBD), immediately downstream from their secretion signal [23–25]. CBDs are extended non-globular polypeptides that wrap around the chaperone, interacting with large hydrophobic regions [25]. This non-stringent mode of interaction could explain the ability of these chaperones to interact with a diverse range of effectors. Chaperones have not been characterized for every TME; however, SscB is the chaperone for both *Salmonella* SPI-2 TMEs SseF and SseG [26], SrcA is the chaperone for *Salmonella* SPI-2 TME SteD [27], CesT is the chaperone for *E. coli* TME Tir [24] and VecA is the chaperone for *Vibrio parahaemolyticus* TME VopQ/VepA [28]. Structural data revealed the nature of the interaction between the N-terminal CBD of Tir and CesT and a second chaperone-binding motif in the C-terminal region of Tir was also identified [29]. However, this structural model was made using small fragments of Tir lacking the TM regions so cannot be used to determine whether they also interact with CesT. Crosslinking experiments show that SscB interacts with both the CBD and the first TM region of SseF, which is the most hydrophobic of its two TM regions [12]. This raises the possibility that interaction with a chaperone could be a general means of protecting the hydrophobic TM regions of injectisome TM substrates from aggregation. Indeed, SteD is insoluble when overexpressed in *E. coli* but forms soluble complexes when co-expressed with SrcA [27]. On the other hand, purified VopQ and Tir are both soluble [30, 31]. Therefore, for both TMEs and translocon proteins, there is evidence that their chaperones protect their TM regions to prevent aggregation in the bacterial cytoplasm.

A systematic study of TM substrates from type three injectisomes (including translocon proteins) revealed that their TM regions are less hydrophobic than canonical single-pass TM regions of bacterial inner membrane proteins but more hydrophobic than soluble cytoplasmic proteins [12]. This intermediate hydrophobicity was found to be permissive for integration into host cell membranes but insufficient for SRP recruitment, which requires a higher density of hydrophobic residues [32]. The first TM region of the *Salmonella* SPI-2 effector SseF was the only individual TM region of three injectisome substrates tested that was able to drive membrane integration by the SRP pathway. However, full-length endogenous SseF, along with other injectisome TM substrates, did not integrate into the bacterial inner membrane when its export was prevented [12]. On the other hand, deletion of the SseF chaperone SscB resulted in bacterial inner membrane integration of SseF [12]. Therefore, in addition to chaperone binding, the relatively low hydrophobicity of their TM regions appears to prevent injectisome TM substrates from recruiting SRP and integrating into the bacterial inner membrane.

## Membrane integration and assembly of the translocon pore

To assemble a translocon pore, both the major and minor translocon proteins must transition from partially unfolded monomers that pass through the narrow channel of the needle, to a membrane-integral oligomer. Based on sequence similarity, T3SS injectisomes from different bacteria can be divided into subfamilies. The highest level of sequence identity of the translocon proteins is found within their transmembrane regions. This, along with their overall evolutionary conservation, suggests that they are likely to share similar mechanisms of membrane integration and assembly.

**Fig. 1.**
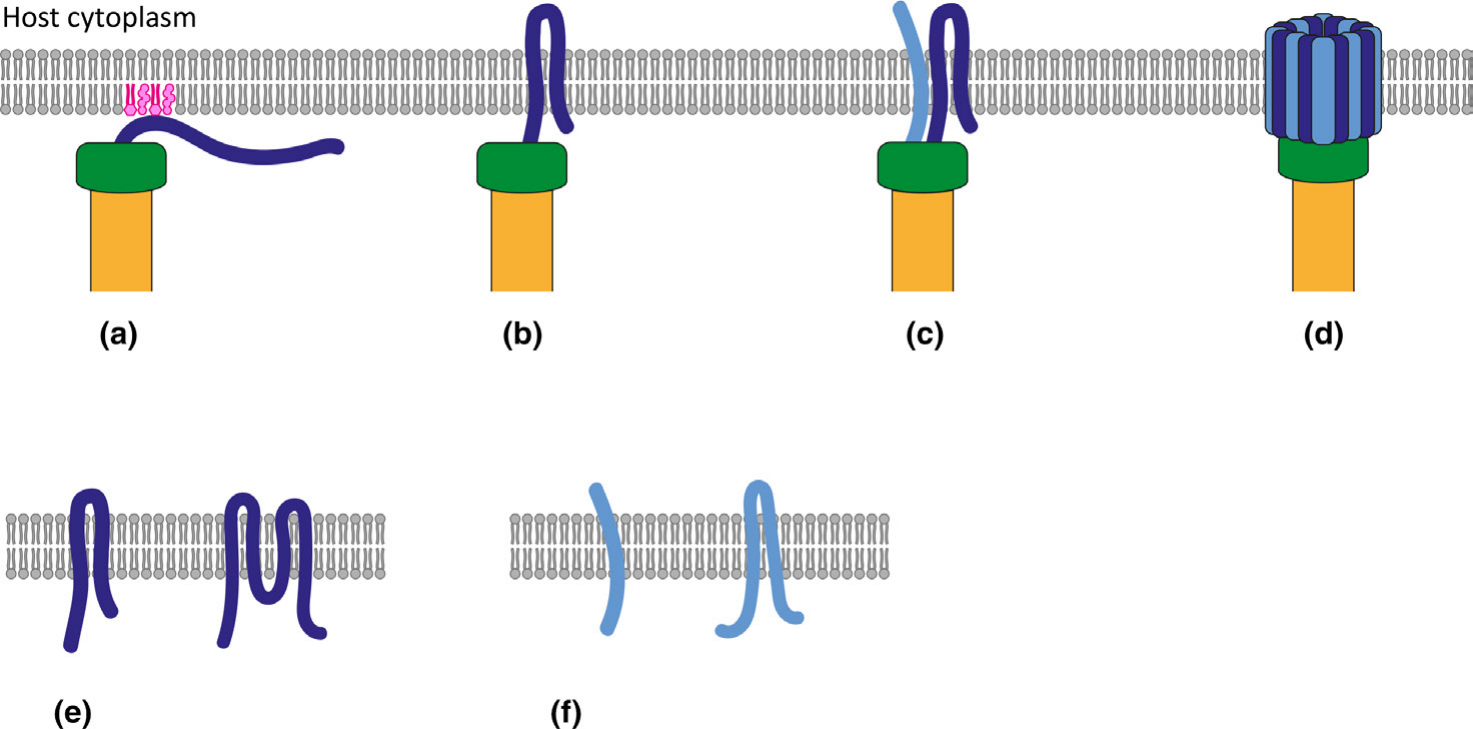
Membrane integration of translocon proteins and assembly of the translocon pore. Major (dark blue) and minor (light blue) translocon proteins pass through the injectisome needle (yellow) as partially unfolded monomers. The hydrophilic ‘tip complex’ (green) provides a scaffold for the assembly of the translocon. (a) Initial peripheral interaction of the C-terminal region of the major translocon with specific lipids (pink), including charged lipid head groups and cholesterol. (b) Insertion of the transmembrane regions of the major translocon into the membrane (grey). (c) Minor translocon membrane integration. (d) Oligomerization and pore formation. (e) Two possible topologies of the major translocon in the membrane. If one helical hairpin integrates into the membrane it would be a two-pass TM protein. If two helical hairpins integrate into the membrane it would be a four-pass TM protein. (f) Two possible topologies of the minor translocon in the membrane. It either has one or two TM regions.

Translocon proteins can integrate spontaneously into artificial liposome membranes and form pores, as demonstrated by inducing secretion of the *Y. enterocolitica* translocon proteins [33] or with purified *P. aeruginosa* translocon proteins [21, 34, 35] in the presence of liposomes. Although both major and minor translocon proteins are required for pore formation [36–38], each can self-integrate in artificial membranes independently of each other [39–43]. However, during infection, the hydrophilic tip complex protein is required for a functional translocon pore [36, 38, 44, 45]. Structural studies have shown that the *Salmonella* SPI-1 hydrophilic protein SipD forms a pentameric ring, whose lumen is continuous with the open channel of the needle [46]. In contrast, the *E. coli* hydrophilic protein, EspA, forms a long, hollow sheath-like filament that links the needle to the translocon pore [47, 48]. An interaction between the hydrophilic protein and the major translocon protein could position the translocon protein to enable integration or alter its conformation by releasing the TM helices for insertion [49]. In either case, the hydrophilic protein is thought to provide a scaffold from which the assembly of the translocon at the tip of the needle is orchestrated (Fig. 1) [50]. In addition, the major translocon proteins of *Shigella* and *Salmonella* SPI-1 injectisomes have been shown to be acylated by a bacterial acyl carrier protein, which promotes but is not absolutely required for pore formation [51, 52].

While no host proteins appear to be required for insertion of translocon proteins into membranes, the lipid composition of the host membrane is important. Depletion of cholesterol from the plasma membrane of cells prevented contact-mediated haemolysis and effector translocation by the *Shigella* and *Salmonella* SPI-1 injectisomes, respectively [53, 54] . The major translocon components of both injectisomes interact with cholesterol [53] and *Salmonella* injectisome-dependent invasion of epithelial cells correlates with cholesterol content and plasma membrane localization over the course of the cell cycle [55]. Contrasting results from *in vitro* reconstitution experiments with purified proteins suggest that the presence of cholesterol in artificial liposomes either aided [21] or was not required [34] for pore formation by the *P. aeruginosa* translocon. The structural relationship between translocon proteins and cholesterol needs to be clarified and more work is required, using physiological membranes, to understand if and how cholesterol mediates translocon protein insertion and assembly in target membranes.

Pore formation in artificial liposomes is more efficient at acidic pH and the presence of negatively charged lipids is required [21, 34, 35, 43], suggesting that ionic interactions between the translocon proteins and lipid head groups are involved at the initial stages of membrane interaction or integration. Indeed, the C-terminal region of the *Salmonella* SPI-1 major translocon protein SipB associates with liposome membranes through a peripheral membrane interaction and deletion of this region prevents membrane integration of the TM regions [42]. This non-TM C-terminal region is predicted to form an amphipathic helix, which could interact with the charged surface of the membrane, thereby explaining the requirement for negatively charged lipids. The C-terminal region of the *A. hydrophila* major translocon protein AopB has also been shown to be required for membrane integration [20]. An initial interaction could function in bringing the major translocon into position at the membrane for subsequent integration. Membrane integration of the major translocon therefore involves two steps: an initial peripheral interaction between the C-terminal region and the membrane followed by integration of the TM regions (Fig. 1).

Protease protection and cysteine substitution mutagenesis studies showed that SipB [42], the *P. aeruginosa* major translocon PopB [56] and the *Shigella* major translocon IpaB [52] have two TM regions, and that the section between them passes through the plasma membrane and forms a loop on the cytoplasmic side. This leaves the N and C terminal regions outside the cell. The only solved structure of a major translocon protein including the TM regions is that of AopB in complex with its chaperone AcrH in solution [20] (see above). This structure reveals two alpha helical hairpins within predicted TM regions, with the first being more hydrophobic than the second. The more hydrophobic hairpin is protected from exposure to solution by hydrophilic alpha helices in AopB and AcrH. This is reminiscent of the structure of the pore-forming domain of colicin Ia, which also spontaneously inserts into membranes [57, 58]. Upon insertion of the most hydrophobic hairpin of colicin Ia, the hydrophobic interactions within the protein are replaced by interactions with the hydrophobic core of the bilayer [59]. Nguyen *et al*. suggest that a similar structural rearrangement drives spontaneous integration of the more hydrophobic helical hairpin of AopB [20]. The associated conformational change could then allow the less hydrophobic hairpin to insert into the membrane. This model could be applicable to major translocon proteins from other injectisomes. However, it is not clear how the helices that make up the AopB hairpins are arranged within the membrane. The helices of the second hairpin, which are 20 and 17 residues long, seem long enough to span the membrane. However, the helices of the more hydrophobic hairpin, which are only 12 residues long, would have to extend upon integration. Assuming that both hairpins integrate into the membrane, the major translocon protein is a four-pass TM protein [20]. Alternatively, it would be a two-pass TM protein if the less hydrophobic hairpin does not integrate (Fig. 1). This distinction affects which residues line the pore. One clear difference between the major translocon and colicins is that colicins transition from a well-folded, soluble state to a membrane integral state, whereas the major translocon disassociates from its chaperone and is at least partially unfolded as it passes through the injectisome needle before membrane integration.

There is some evidence to suggest that stable integration of the minor translocon protein is dependent on the initial integration of the major translocon (Fig. 1) [37, 56]. Upon integration, the N- and C-termini of minor translocon proteins IpaC from *Shigella* and SipC from *Salmonella* SPI-1 have been shown to be on opposite sides of the membrane, suggesting that minor translocons only have one TM region [39, 60]. However, contradictory data suggest that both the N- and C-termini of *Shigella* IpaC and *A. hydrophila* AopD are left outside the plasma membrane after membrane integration [20, 61], suggesting that they have two TM regions (Fig. 1).

Within the membrane, the major and minor translocon components interact to form a heteromeric complex [62–64] with a toroid ring-like structure (Fig. 1) [4, 21]. Initial work to determine the stoichiometry of the translocon within the membrane suggests that the complex consists of 15–20 YopB/D subunits for *Yersinia* [63] and 8 PopB and 8 PopD subunits for *Pseudomonas* [64]. This leads to a pore in the membrane that has been reported to have an inner diameter of ~ 2–4 nm [65]. Therefore, it seems likely that the major and minor translocon proteins assemble into a 16-subunit complex with a 1 : 1 stochiometry to form the translocon pore. High-resolution structural studies of translocons *in situ* are required to determine how the translocon proteins are arranged in the membrane to form a pore.

## Membrane integration of TMEs

In contrast to the translocon proteins, TMEs are varied in both sequence and function (Table 2). However, most TMEs discussed in this review have, or are predicted to have, two TM regions and integrate such that the section between the TM regions passes through the membrane and forms a loop on the luminal side, leaving both the N- and C-terminal regions in the host cytoplasm. This includes the *Salmonella* SPI-2 TMEs SteD, SseF and SseG and the *E. coli* TMEs, Tir, EspZ and NleA/EspI. It also includes approximately 50 *Chlamydia* TMEs known as Incs that integrate into the membrane of the *Chlamydia* inclusion body [66]. Indeed, this shared membrane topology has made it possible to predict new Incs by searching the *Chlamydia* genome for proteins with two alpha helices of 15–32 residues connected by a short section of 3–22 residues [66–68]. The transmembrane regions of other effectors can be identified using *in silico* prediction software. Their ability to integrate into membranes should then be confirmed using biochemical fractionation assays. Interestingly, the sections between the two TM regions are enriched in residues that have a high propensity to induce a turn between two alpha helices, which could explain the topology of these proteins [66, 69]. The shared topology of TMEs also suggests similarities in the mechanisms of membrane integration.

**Fig. 2.**
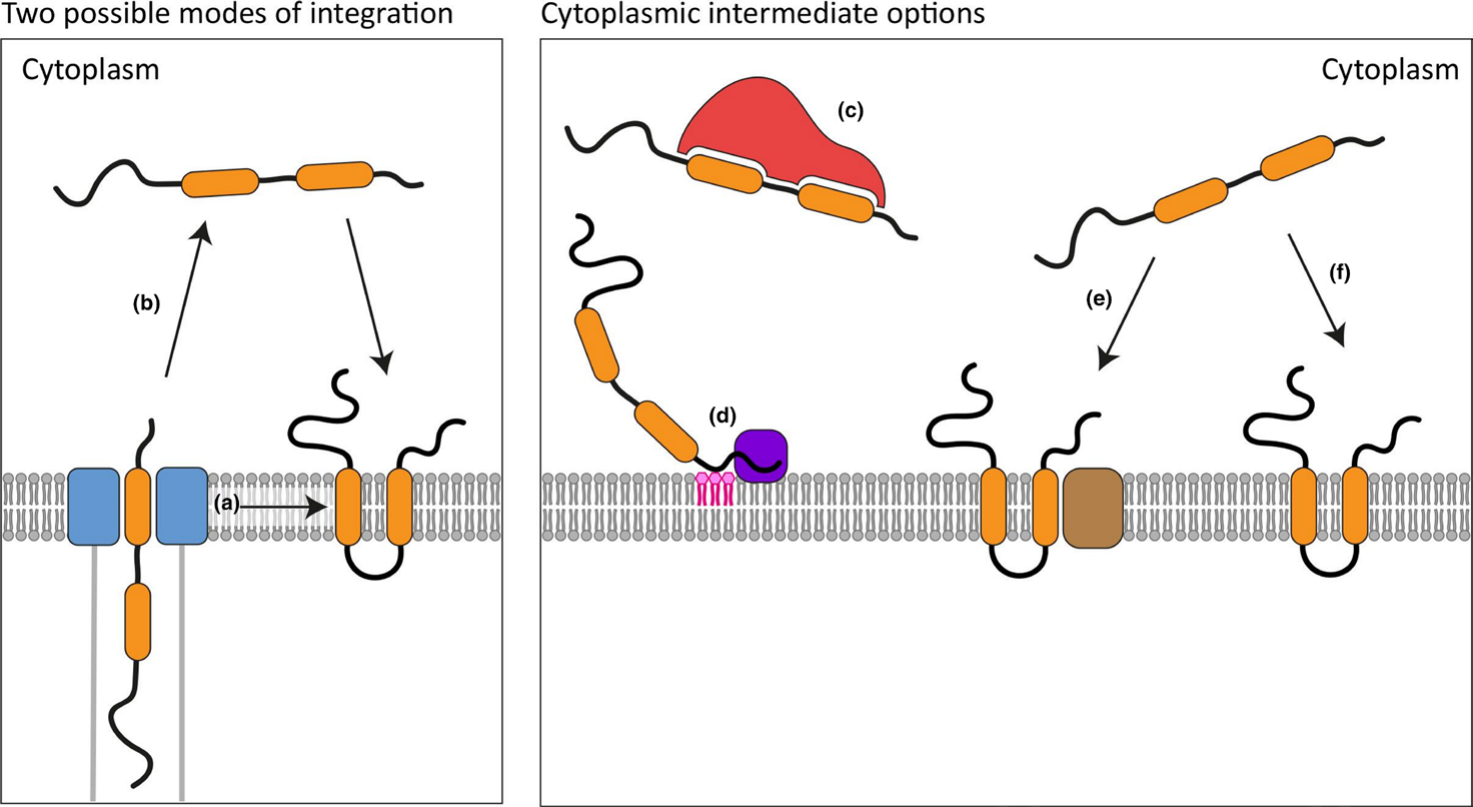
Potential mechanisms of transmembrane effector membrane integration. Transmembrane effectors (orange) could enter mammalian membranes (grey) either by (a) passing laterally through the translocon pore (blue) or (b) passing through a cytoplasmic intermediate stage before integration. From the cytoplasm, several different integration mechanisms are possible. (c) The transmembrane effector might require interaction with a host chaperone (red) to avoid aggregation. (d) An initial interaction with a specific lipid (pink) or host membrane protein (purple) might position the transmembrane effector at the relevant membrane for integration. (e) Membrane integration might be protein-assisted using an insertase-like protein (brown). (f) Autonomous membrane integration.

There are two possible pathways for integration of TMEs into host cell membranes. They could integrate into the membrane containing the injectisome translocon, followed by retention in that membrane or budding from the membrane and vesicular trafficking to a target organelle (Fig. 2a). For this to occur, a mechanism analogous to that of the endoplasmic reticulum (ER) or SecYEG translocon might be possible. Structural models and biochemical interaction data have indicated how a lateral gate in these translocons opens to create a direct interface between the protein channel and the hydrophobic interior of the membrane, allowing proteins to pass directly into the membrane [70, 71]. However, there are currently no data to support this model for the injectisome translocon. Alternatively, TMEs could be delivered into the host cytoplasm before integrating into the relevant membrane (Fig. 2b). In support of the cytoplasmic intermediate model, TMEs including Tir, SteD, SseF, SseG and VopQ integrate into membranes when expressed ectopically in eukaryotic cells [72–75]. This shows that their membrane integration does not require the injectisome translocon or any other bacterial proteins.

Class I effector chaperones are not delivered into the host cell [16], so if the cytoplasmic intermediate model is correct, TMEs might require interaction with a host chaperone to prevent their aggregation in the host cytoplasm (Fig. 2c). Alternatively, it is possible that the relatively low hydrophobicity of the TM regions of TMEs [12] allows them to remain soluble in the cytoplasm of the host cell before they reach their target membrane. Indeed, VopQ transitions from a soluble to a membrane-inserted state [75].

TMEs are found at distinct membrane compartments, showing that their localization is regulated. Many are localized in the membrane that contains the injectisome translocon: e.g. Tir is found in the plasma membrane, SseF and SseG in the SCV membrane and Incs in the *Chlamydia* inclusion membrane [76–78]. In contrast, following its delivery through the SCV membrane, the *Salmonella* TME SteD is found in the *trans*-Golgi network (TGN) and in endosomes including major histocompatibility complex (MHC)II compartments [73]. The *V. parahaemolyticus* TME VopQ is found at endolysosomal compartments [75]. The *E. coli* TMEs Tir and NleA/EspI are delivered through the plasma membrane. However, NleA/EspI localizes at the Golgi and is recruited there through interaction with the COPII component, Sec24 [79, 80]. Tir is mainly found at the plasma membrane, but also at the Golgi network [81]. Blocking secretory traffic prevented plasma membrane localization of Tir, suggesting that Tir first integrates into the Golgi membrane from the cytoplasm and then uses the secretory pathway for traffic to the plasma membrane [81].

An initial interaction with a specific lipid headgroup or membrane protein could position TMEs at the relevant membrane for integration and therefore determine localization (Fig. 2d). *In vitro* reconstitution work on the integration of Tir into artificial liposome membranes indicated that Tir has a preference for liposomes containing sphingomyelin [31]. Two alternative models for a two-step process of Tir binding and integration were suggested: either binding of the C-terminal region to the membrane surface followed by insertion of the TM regions, or vice versa. Mutational analysis of SteD demonstrated that its localization at the Golgi can be uncoupled from membrane integration, suggesting that an interaction with a membrane component (either lipid or protein) is the first step in membrane integration [82]. The ability of VopQ to interact with artificial membranes *in vitro* is dependent on the presence of negatively charged lipids at acidic pH, which enables an electrostatic interaction [75]. However, at physiological pH and *in vivo*, the association of VopQ with membranes depends on its interaction with the V-ATPase complex. This restricts the membrane association of VopQ, and therefore its integration, to membranes containing the V-ATPase [75]. It therefore seems that a two-step process of membrane recruitment and integration might be a common mechanism for TMEs.

Do TMEs require host protein assistance, for example an insertase-like protein, to integrate into the membrane (Fig. 2e)? *In vitro* reconstitution work showed that Tir integrates spontaneously into liposome membranes in the absence of any additional proteins (Fig. 2f) [31]. In addition to the translocon proteins discussed above, other proteins, for example pore-forming toxins [57] and some thylakoid integral membrane proteins [83], insert spontaneously into membranes. To date, no mammalian proteins have been shown to be required for TME integration, but this possibility deserves further investigation.

## The roles of injectisome TM substrates in pathogenesis

By positioning themselves within a membrane, TMEs can have a range of biochemical functions that are not available to cytoplasmic effectors.

### Translocon proteins

As well as forming the translocon pore, translocon proteins from some bacterial pathogens have additional functions that aid bacterial virulence. Some *Salmonella* SPI-1 effectors promote internalization of bacteria by inducing actin cytoskeleton rearrangements. These lead to membrane ruffling, trapping of bacteria and their uptake. In addition, the C-terminal cytoplasmic region of the *Salmonella* SPI-1 minor translocon SipC nucleates and bundles actin [84, 85]. The SPI-1 effector SipA enhances this activity by stabilizing actin filaments [86, 87]. While SipC is not absolutely required for these rearrangements, its activity makes bacterial uptake more efficient by concentrating actin filaments at the translocon pore, where the bacterial cell interacts with the host plasma membrane.

The *Shigella* homologue of SipC, IpaC, also promotes actin polymerization at the bacterial/host cell contact point. However, it does this by recruiting the tyrosine kinase Src rather than through direct actin nucleation [88]. SipC has also been shown to interact with intermediate filaments, which promotes effector translocation [89].

### Intramembrane interactions

**Fig. 3.**
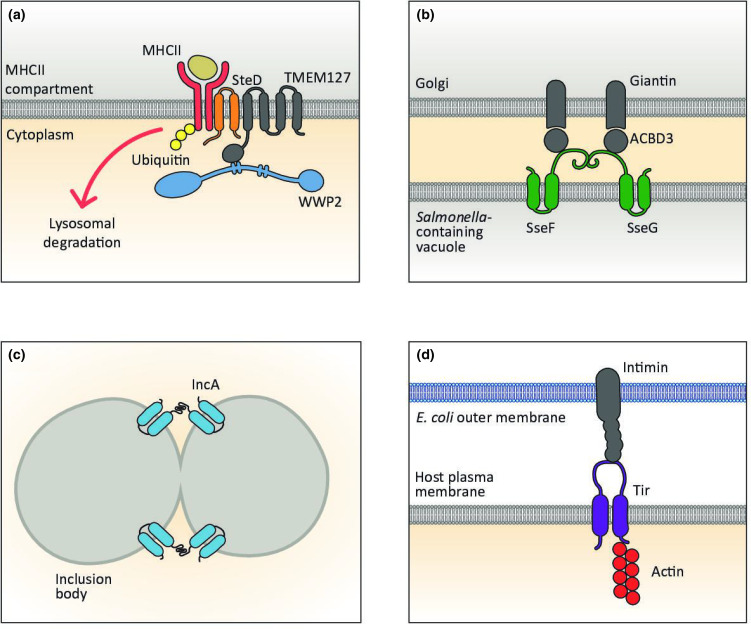
Examples of transmembrane effector functions. (a) The *Salmonella* transmembrane effector SteD forms an intramembrane complex with TMEM127. This recruits the host E3 ligase WWP2, resulting in ubiquitination of MHCII and its lysosomal degradation. (b) The *Salmonella* transmembrane effectors SseF and SseG anchor the *Salmonella *-containing vacuole to the Golgi. This membrane contact is mediated by interactions between SseF, SseG and the peripheral Golgi protein ACBD3. ACBD3 interacts with the Golgi membrane-integrated protein, giantin. (c) The *Chlamydia* transmembrane effector IncA mimics a eukaryotic SNARE protein, leading to homotypic fusion of membrane compartments and the formation of one large inclusion body containing *Chlamydia* cells. (d) The *E. coli* transmembrane effector Tir functions as a receptor in the plasma membrane of host cells, by interacting with intimin on the surface of *E. coli* cells. This establishes close contact between the bacteria and the surface of the host cell.

The TM regions of TMEs can interact with TM regions of other proteins to form complexes within the membrane. This kind of interaction is important for the function of the *Salmonella* SPI-2 injectisome TME, SteD (Fig. 3a). SteD functions as an adaptor, through an intramembrane interaction with a TM protein called TMEM127 [90]. TMEM127 contains a canonical PPxY motif at its cytoplasmic C-terminus, through which it interacts with the NEDD4-like E3 ubiquitin ligase, WWP2. WWP2 then induces the ubiquitination and lysosomal degradation of at least three targets of SteD that are required for immunological synapse formation and antigen presentation – CD97 [91], CD86/B7.2 and MHCII [73]. This results in their reduction from the surface of antigen-presenting cells and a disruption of the adaptive immune response.

### Linking membrane compartments

TM proteins are also involved in the formation of membrane contact sites, which are important for holding membranes in close apposition without membrane fusion [92], facilitating non-vesicular transport of lipids, ions and other molecules between the two membrane compartments. Two *Salmonella* SPI-2 TMEs, SseF and SseG, are necessary for close association between the SCV and the Golgi network in epithelial cells (Fig. 3b) [74, 93, 94]. Both proteins integrate into the SCV membrane, where they hetero-oligomerize [78, 95]. Deletion of either prevents the Golgi localization of SCVs and causes a *Salmonella* replication defect after infection [93]. Both TMEs interact with ACBD3, a Golgi-resident protein, which is also required for SCV association at the Golgi network [96]. In this way, the interaction of SseF, SseG and ACBD3 is proposed to form a tether between the SCV and the Golgi, which could be necessary for the acquisition of nutrients for *Salmonella* growth.

The *Chlamydia* TMEs IncV and IncD have redundant functions in the formation of membrane contact sites between the inclusion body membrane, in which they integrate, and the ER [97]. They do this through interaction with the ceramide transfer protein CERT and its TM ER binding partners VAPA/B. These proteins are commonly present at ER–Golgi contact sites, where they facilitate non-vesicular transport of ceramide between the ER and the Golgi [98]. IncV has two FFAT motifs (two phenylalanines in an acidic tract), which interact with VAPA/B [97], and IncD interacts with the pleckstrin homology (PH) domain of CERT [99] These interactions tether the inclusion membrane to the ER [99–101] and are required for the ceramide transport to the inclusion and its subsequent conversion to sphingomyelin, which is important for *Chlamydia* replication [102].

### Membrane fusion through SNARE activity

The large *Chlamydia* inclusion body forms as a result of homotypic fusion between individual inclusion bodies [103]. Disrupting the *Chlamydia* TME IncA results in aberrant multilobed inclusion structures [104, 105], thereby implicating it in homotypic fusion (Fig. 3c). In eukaryotic cells SNAREs [soluble NSF (N-ethylmaleimide-sensitive factor) attachment protein receptors] are the main regulators of membrane fusion. SNAREs are anchored into membranes either by TM domains or through lipidation and they either drive or inhibit membrane fusion through coiled-coil formation with SNAREs in opposing membranes [106]. IncA has two SNARE-like domains (SLDs) in its C terminal cytoplasmic domain [107, 108]. IncA self-interacts and these SLDs are both required for homotypic fusion of *Chlamydia* inclusion bodies, indicating that IncA mimics eukaryotic SNAREs [109, 110]. Interestingly, although they fuse together to form large inclusion bodies, inclusions are non-fusogenic with endocytic and lysosomal compartments [111]. The IncA SLDs also interact with SNAREs from the endosomal pathway (Stx7, Stx8m Vtilb and VAMP8) and recruit them to the inclusion membrane [108]. However, IncA prevents the fusion of liposomes containing these SNAREs. Thus, IncA facilitates homotypic fusion through self-interaction, and additionally acts as an inhibitory SNARE in complex with endosomal SNAREs, which enables *Chlamydia* to avoid lysosomal degradation.

### Membrane disruption

The *V. parahaemolyticus* T3SS-1 TME VopQ contributes to cytotoxicity by inducing autophagy [112]. VopQ penetrates membranes following interaction with the V_o_ subcomplex of V-ATPase [75, 113]. It has been suggested that it forms pores, leading to the release of ions from the lysosome and its deacidification. The structure of VopQ in complex with the V_o_ subcomplex has been solved by cryo-EM [30]. It suggests that charged amino acids of VopQ’s three TM helices contact the hydrophobic core of the membrane and create local instability. However, the TM residues were disordered and not resolved, so the exact mechanism of membrane disruption remains unclear.

### Regulation of membrane trafficking

The *E. coli* TME NleA/EspI, which localizes at the Golgi, interacts with Sec24, a component of the COPII vesicle coat [79]. This interaction stabilizes COPII at the Golgi, resulting in a decrease in protein secretion [114, 115]. This disrupts MHCII invariant chain transport and might therefore affect antigen presentation [115]. However, due to the general nature of this effect, the physiological significance of disrupting protein secretion on *E. coli* infection is not clear.

Although the functions of many Inc proteins in different *Chlamydia* species remain unknown, some have been implicated in the recruitment of host trafficking proteins, including Rab proteins and sorting nexin (SNX) proteins to the inclusion membrane. However, it is not clear whether or how integration into the membrane is required for this function. The Rab family are the largest branch of the Ras superfamily of small GTPases and are involved in membrane traffic through recruitment of Rab effectors. By recruiting Rabs, *Chlamydia* effectors regulate membrane trafficking to the inclusion to promote nutrient uptake and survival. The *Chlamydia trachomatis* Inc CT229 recruits Rab4 to the inclusion membrane [116] and the *Chlamydia pneumoniae* Inc Cpn0585 recruits Rab1, Rab10 and Rab11 to the inclusion membrane [117]. Interestingly, the Rabs that are found at the inclusion membrane are those involved in retrograde trafficking and biosynthetic pathways, whereas those normally involved in controlling transport along the phagocytic pathway are excluded from the inclusion membrane [111]. This, along with the selective membrane fusion controlled by IncA, goes some way toward explaining how the inclusion membrane avoids lysosomal fusion to create a suitable niche for *Chlamydia* growth and replication.

SNX proteins are components of the retromer complex, which is involved in the traffic of receptors from endosomes to the TGN. SNX proteins consist of a PX domain, which interacts with phosphoinositide lipids and a Bin-amphiphysin-Rvs (BAR) domain, which senses and induces membrane tubulation, leading to the formation of vesicles. *C. trachomatis* IncE recruits SNX5/6 to the inclusion via interaction with their PX domains, causing membrane tubulation [118]. However, depletion of SNX5/6 by RNAi increases infectious progeny, suggesting that this regulation of membrane trafficking by IncE is inhibitory to bacterial growth. Other Incs recruit proteins to the inclusion membrane, suggesting that this is a major role for Incs [119].

### Transmembrane signalling

By spanning membranes, TMEs have access to two physically separated compartments, which allows communication between them. *E. coli* has at least two TMEs that are receptors at the plasma membrane. EspZ localizes at sites of bacterial attachment in the plasma membrane [120, 121]. Following transfection, it is also found at mitochondria, but it is unclear if this location is relevant to its function [122]. EspZ limits the level of effector translocation into the cell and therefore protects the cell from rapid cell death following *E. coli* infection, implicating EspZ as a pro-survival effector [120, 122, 123]. Although the mechanism of its action is not fully understood, its extracellular loop provides EspZ with strain specificity. This could suggest that it interacts with bacterial proteins on the cell surface or part of the injectisome machinery.

The extracellular loop of plasma membrane-localized Tir interacts with the *E. coli* protein intimin, which is expressed on the bacterial cell surface [76, 124]. This interaction leads to clustering of Tir in the membrane, phosphorylation of a tyrosine residue in its C-terminal cytoplasmic tail and activation of actin polymerization [72], resulting in actin pedestals at the site of *E. coli* attachment [125]. The interaction between Tir and intimin leads to the tight attachment of *E. coli* to the surface of the host cell (Fig. 3d) [126]. Therefore, both *E. coli* TMEs transmit information from outside the cell into the infected cell cytoplasm.

## Concluding comments and future directions

The TM substrates of T3SS injectisomes of bacterial pathogens share the ability to integrate into host membranes while avoiding both integration into the bacterial inner membrane and aggregation. Their position within membranes enables a distinct range of functions, including pore formation, mediating intramembrane interactions, signalling and linking membrane compartments, which all depend on interactions with other proteins. Interestingly, no TMEs studied to date appear to function as enzymes, in contrast to many eukaryotic TM proteins [127] and the majority of soluble injectisome effectors [128].

Translocon proteins and TMEs have different membrane integration mechanisms but neither are fully understood. Translocon proteins transition from an unfolded to a membrane integral state and self-assemble to form large, stable heteromeric complexes in the membrane. This integration mechanism might share evolutionary ancestry with colicins [20]. The assembly of the translocon pore within membranes requires an additional interaction with a translocon-associated hydrophilic protein. Recent advances in cryo-electron microscopy have enabled progress in the structural determination of the T3SS injectisome [129]. Despite some information on the structure of the translocon pore [4], the finer molecular details remain to be solved. Additional structural studies will help determine how the interaction between the hydrophilic component and the translocon enables translocon assembly and how well conserved this mechanism is between different injectisomes. They will also allow confirmation of the stochiometry, topology and arrangement of the translocon pore.

Structural determination of TMEs is inherently difficult due to their insolubility in solution. This could be aided by the large advances in structural prediction delivered by AlphaFold [130]. It is not clear whether, like translocon proteins, TMEs oligomerize in membranes and, if so, what the functional significance of oligomerization is. In contrast to translocon proteins, membrane integration of TMEs does not appear to require any additional bacterial proteins. However, it is not clear whether the ability to target and integrate into membranes is an inherent property shared by these proteins or if they require assistance from host proteins - as chaperones, for membrane targeting or for integration (Fig. 2). It is essential to establish if TMEs are present in the host cytoplasm before integration and, if so, whether their stability depends on a cytoplasmic chaperone. Structural studies of the injectisome translocon pore as well as crosslinking experiments of TMEs during translocation could clarify whether TMEs pass laterally into the membrane during translocation or if they are delivered into the host cytoplasm. Single-molecule studies of TMEs could describe their progression from the injectisome to the host cell membrane and whether they pass through the cytoplasm. Knockout screens will be useful to identify whether any eukaryotic proteins are involved in TME membrane targeting and integration.

If Tir’s ability to integrate spontaneously occurs *in vivo* and is shared by other effectors, this could circumvent any requirement for host protein-aided integration. In this case, integration might depend on the specific membrane composition, as seen for Tir and translocon proteins, whose integration is stimulated by and dependent on sphingomyelin and cholesterol, respectively [31, 53]. It is important to determine whether other TMEs have different lipid requirements for integration.

The specific localization of TMEs suggests that they integrate at distinct membrane compartments. A mechanism analogous to that of translocon proteins could exist, in which a specific interaction positions TMEs for subsequent integration. The interaction between VopQ and the V-ATPase provides an example of such a process. In the case of SteD, it remains to be determined which host components it interacts with at the ER or Golgi and what role this interaction has in integration. The evidence indicating that Tir is first targeted to the Golgi before transfer to the plasma membrane needs to be confirmed and further investigated, which could be done using kinetic microscopy assays.

Integration of TMEs leaves portions of the proteins exposed to the cytoplasm, which are accessible for post-translational modification, for example ubiquitination of SteD [90] and phosphorylation of Tir [72]. The N-terminal tail of SteD also contains a sequence that resembles an inverted signal sequence and allows it to hijack a host vesicular trafficking pathways from the TGN to reach MHCII compartments [82]. More work is required to establish the full range and functional significance of post-translational modifications and interactions conferred by the cytoplasmic sections of TMEs.

The importance of TMEs in *E. coli *, *Salmonella* and *Chlamydia* pathogenesis indicate that membrane integration of effectors is a common pathogenic mechanism. Protein identification from purified membrane fractions following infection could help identify new TMEs and confirm the presence of TM regions predicted by *in silico* software. This kind of analysis is required to determine how widespread TMEs are. Finally, functional characterization of this understudied group of effectors will classify what additional pathogenic mechanisms are conferred by their position within membranes.
